# Mental disorder and PTSD in Syria during wartime: a nationwide crisis

**DOI:** 10.1192/bjo.2021.696

**Published:** 2021-06-18

**Authors:** Sami Jomaa, Ameer Kakaje, Ragheed Al Zohbi, Osama Hosam Aldeen, Leen Makki, Ayham Alyousbashi, Mhd Bahaa Aldin Alhaffar

**Affiliations:** 1 Faculty of Medicine, Damascus University; 2 Faculty of Medicine, Aleppo University; 3 Faculty of Medicine, Aleppo University, Department of Experimental Surgery, McGill University; 4 Department of Periodontology, Faculty of Dentistry, Damascus University

## Abstract

**Aims:**

Syria has experienced war since 2011, leaving over 80% under the poverty line and millions displaced. War and its retaliations have significantly impacted the mental health of Syrians. This study evaluates the post-traumatic stress disorder (PTSD), and the severity of the mental distress caused by war and other factors such as low social support. This study also evaluates other variables and compares the findings with those of multiple studies on Syria and refugees.

**Method:**

This is a cross-sectional study that included people who lived in Syria in different governorates. Online surveys were distributed into multiple online groups and included the Kessler 10 (K10) scale which screens for anxiety and depression, the Screen for Posttraumatic Stress Symptoms (SPTSS) tool, the Multidimensional Scale of Perceived Social Support, and questionnaires on demographic and war-related factors.

**Result:**

Our study included 1951 participants, of which, 527 (27.0%) were males and 1538 (78.8%) between the age of 19 and 25. Among participants, 44% had likely severe mental disorder, 27% had both likely severe mental disorder and full PTSD symptoms, 36.9% had full PTSD symptoms, and only 10.8% had neither positive PTSD symptoms nor mental disorder on the K10 scale. Around 23% had low overall support. Half of the responders were internally displaced, and 27.6% were forced to change places of living three times or more due to war. Around 86.6% of the responders believed that the war was the main reason for their mental distress. Those with high SPTSS and K10 scores were found to take more days off from work or school due to negative feelings and having somatic symptoms. Moreover, the number of times changing places of living due to war, educational level, and being distressed by war noise were the most prominent factors for more severe PTSD and mental distress. No differences in PTSD and mental disorder prevalence were noted in participants living in different governorates or among different types of jobs. A strong significant correlation (r = 0.623) was found between SPTSS and K10 scores.

**Conclusion:**

The conflict in Syria has left the population at great risk for mental distress which was higher compared to Syrian refugees elsewhere. Many measures with an emphasis on mental health are needed to help the people against a long-term avoidable suffering.

